# The Solvency of Approved Societies

**Published:** 1914-04-04

**Authors:** 


### THE EDITOR'S LETTER-BOX.

### The Solvency of Approved Societies.

*To the Editor of* The Hospital.

Sir,—Your account of the Fabian Society's interim report on the working of the Insurance Act is of especial interest, in that, as you point out, the Fabians are, if anything, biassed in favour of the Act rather than against it. For this reason the conclusions of that Society's Committee of Inquiry, where they are of the nature of criticism, may be taken as the irreducible minimum of the Act's deficiencies. The financial standing of the Act, or, rather, of the Approved Societies, is probably the most important problem of the moment in National Insurance. It is obvious that the Fabians are by no means sure that these societies are solvent, and, indeed, are certain that a good many of them are insolvent. This important question of fact, or of actuarial valuation, has unfortunately become the sport of party politicians. The Opposition naturally take a gloomy view of what the valuations next year will disclose; the supporters of the Government are in their turn as optimistic as their opponents are the reverse.

In these circumstances the truth is likely to lie, as the Fabians assert that it does, somewhere betwixt and between. Some societies, doubtless, are easily and triumphantly solvent; some are already hopelessly involved; and some are just keeping their heads above water, with nothing much to spare if a wave of adversity should buffet them. The difference is largely a question of good management; and management is to some considerable degree a question of checking the work-shyness of those who have been drawing benefit, and the downright malingering of those who can humbug their panel doctor. About six months ago the appointment of a number of medical referees in different districts of London disclosed that these two classes contain considerable numbers of individuals; and the Approved Societies were saved much loss by the work of these referees. The referees were remunerated, if I remember rightly, at the rate of seven shillings a case, which rate the bulk of the medical profession considered to be inadequate to the labour and responsibility involved. However, the terms attracted some really good men, and good work has been done by them.

I am informed on unimpeachable authority that two or three weeks ago notices were sent out to them that all available funds were exhausted and that their work would cease the next day! The appointments, as originally made, were only provisional, otherwise such an extraordinary course would not, I suppose, be legal. But the light that is cast by this incident on the financial position of the Act is very significant. Here are medical referees undertaking very successfully the detection of malingering and saving the societies large sums weekly in sick benefits. They accept for this almost nominal fees, which the bulk of the profession regards as inadequate. Yet in *less than six months* they are informed that no more money is forthcoming, and their work therefore ceases. The policy is penny wise, pound foolish, in all probability; yet the Act in London is so bankrupt that even the pennies cannot be raised that will later on save the pounds. It were to be wished that the Fabian Society would investigate this ominous occurrence and discuss it in the final report, which will presumably follow the interim report now presented.—Yours faithfully, Medical Officer.

